# High-Resolution Imaging Reveals New Features of Nuclear Export of mRNA through the Nuclear Pore Complexes

**DOI:** 10.3390/ijms150814492

**Published:** 2014-08-20

**Authors:** Joseph M. Kelich, Weidong Yang

**Affiliations:** Department of Biology, Temple University, Philadelphia, PA 19122, USA; E-Mail: tuf27658@temple.edu

**Keywords:** mRNA, nuclear export, nuclear pore complex, super-resolution microscopy, single-molecule tracking

## Abstract

The nuclear envelope (NE) of eukaryotic cells provides a physical barrier for messenger RNA (mRNA) and the associated proteins (mRNPs) traveling from sites of transcription in the nucleus to locations of translation processing in the cytoplasm. Nuclear pore complexes (NPCs) embedded in the NE serve as a dominant gateway for nuclear export of mRNA. However, the fundamental characterization of export dynamics of mRNPs through the NPC has been hindered by several technical limits. First, the size of NPC that is barely below the diffraction limit of conventional light microscopy requires a super-resolution microscopy imaging approach. Next, the fast transit of mRNPs through the NPC further demands a high temporal resolution by the imaging approach. Finally, the inherent three-dimensional (3D) movements of mRNPs through the NPC demand the method to provide a 3D mapping of both transport kinetics and transport pathways of mRNPs. This review will highlight the recently developed super-resolution imaging techniques advanced from 1D to 3D for nuclear export of mRNPs and summarize the new features in the dynamic nuclear export process of mRNPs revealed from these technical advances.

## 1. Introduction

The presence of the double-membrane nuclear envelope (NE) in eukaryotic cells provides a physical barrier that transcribed mRNA generated in the nucleus must overcome to reach the cytoplasm, whereupon translation can occur on ribosomes generating the corresponding proteins encoded by these transcripts [1,2,3]. Prior to nuclear export, mRNA must first be processed by 5' capping, intron splicing, and polyadenylation [4,5,6,7,8,9,10,11,12]. In addition, mRNA must become packaged into messenger ribonucleoprotein particles (mRNPs) comprising mRNA as well as adaptor proteins and export factors required for efficient nuclear export [13,14,15,16]. Although a recent study has suggested a membrane budding approach for mRNP nuclear export [17], the dominant pathway of mRNP exiting the nucleus is still gated by nuclear pore complexes (NPCs) embedded in the NE.

Two distinct categories of nucleocytoplasmic transport have been identified: passive diffusion and facilitated translocation. The former is reserved for signal-independent diffusing molecules that are smaller than 40 kDa, and the latter ensures efficient and timely translation of signal-dependent cargo molecules assisted by transport receptors through the NPC [18,19,20,21,22]. Moreover, passively diffusing molecules take a transport route consistent with a size-confinement mechanism through a central axial channel [20,23,24]. On the other hand, the facilitated translocation involves extensive interactions between transport receptors and intrinsically disordered nucleoporins (Nups) rich in phenylalanine-glycine (FG) repeats. These FG-Nups comprise one third of all Nups of the NPC and together create a selectively permeable entropic barrier in arguably a form of “polymer brush” or a form of “hydrogel meshwork” for transiting macromolecules [19,22,23,24,25,26,27,28,29,30,31,32,33]. Human mRNPs are large particles with sizes up to 100 MDa [21] that generally require the transport receptor Tap forming a heterodimer with the cofactor p15 to chaperone them through the NPC via interactions between Tap-p15 and the FG Nups [32,33,34]. Furthermore, several other protein complexes have been shown to regulate the human mRNA export process through differing mechanisms including regulation by the transcription and export complex (TREX), and the more recently discovered TREX-2 complex (by its scaffold protein GAMP), both of which function to couple transcription of intron-less, and intron containing mRNAs to nuclear export [35,36,37,38]. The multi-subunit THO complex (THO) is a component of TREX and has been pinpointed as a requirement for nuclear export of polyadenylated RNA, spliced mRNA, mRNA coding for proteins involved in hematopoiesis, as well as for regulation of pluripotency gene mRNA export [39,40]. An additional mechanism has been discovered involving inositol polyphosphate multikinase (IPMK), which regulates transcript-selective nuclear mRNA export to retain genome integrity in humans [41].

## 2. A High-Resolution Microscopy Imaging Approach Is Needed to Look Inside the Nuclear Pore Complex (NPC)

**Figure 1:**
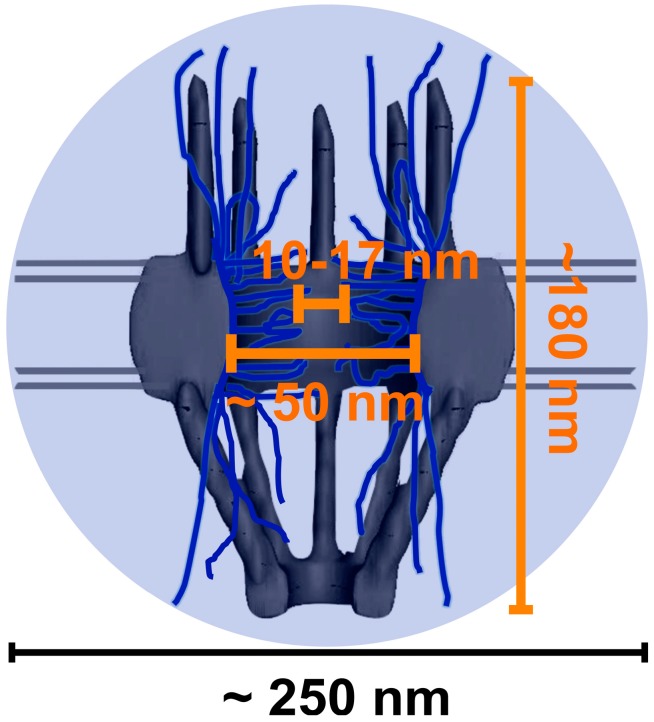
Cartoon image of a nuclear pore complex depicting its length and central axial channel (orange lettering) [42,43,44,45]. Both the nuclear pore complexes (NPC) and the axial channel are below the size of point spread function (PSF) (~250 nm in *x* and *y* plane) of conventional light microscopy. The PSF is represented by the light-blue circled area [20,23,45,52,53,54,55].

Electron microscopy (EM) has revealed the physical structure of the NPC prepared from the chemical-fixed or frozen cell samples. In detail, the NPC is approximately (~180 nm) in length, consisting of the cytoplasmic fibrils (~50 nm), the central scaffold (~40 nm) and the nuclear basket (~90 nm) (Figure 1) [42,43,44,45,46]. Moreover, EM has enabled people to visualize details of mRNPs in the NPC due to its high spatial resolution of ≤1–5 nm [47,48]. Early imaging of salivary gland tissue of *Chironomus tentans* led to descriptions of “balbiani rings”, through which mRNPs were localized and visualized [49,50,51,52]. Additionally, EM data have shown that these mRNPs were elongated during their interaction with the NPC and exported as a linear molecule through the NPC contrasting with the “puff” or “ring” shape of mRNPs observed at other stages of the export process [21,50,53,54]. While helpful in obtaining information from static visualizations of mRNPs in fixed or frozen cells, EM still lacks the *in vivo* capabilities and temporal resolution necessary to capture accurate export kinetics and real-time pathways for mRNPs through NPCs [55,56,57].

Fluorescence microscopy has the advantage of imaging substrates with site-specific fluorescent tags within living cells. Spatial distributions and dynamics of bulk mRNPs labeled by fluorophores in live cells have been well studied using fluorescence microscopy. But the real-time export details of mRNPs in the NPCs of live cells remain largely unknown because of the NPC’s sub-micrometer size. Although it is extremely large relative to the majority of cellular structures, the NPC is still barely below the diffraction-limit point spread function (PSF, ~250 nm in *x* and *y* plane and ~750 nm in *z* dimension) of conventional light microscopy (Figure 1) [58,59,60,61]. Furthermore, the central axial channel of the NPC that ranges from 10 to 17 nm in diameter falls even farther below this diffraction limit [23,42,43,44,45]. Fortunately, single-molecule tracking and super-resolution microscopic technology have recently been developed breaking the limitations of light diffraction of conventional fluorescence microscopy and allowing for super-resolution imaging. So far, several different single-molecule super-resolution fluorescence microscopy approaches have been successfully been applied to elucidate the transport kinetics and spatial routes of mRNPs in live cells (Table 1). These new methodologies have added for the first time the real-time export dynamic pictures of single mRNP molecules within the native NPCs into the static diagram of NPC structure obtained by EM, forming a comprehensive story for mRNP export through the NPC.

**Table 1:** Overview of single-molecule techniques applied to mRNA nuclear export.

Imaging Method	Cell Type	RNA Type (Size in kb)	Illumination Area	Spatiotemporal Resolution	Major Conclusions	Reference
Wide-field epi-fluorescence microscopy	U2OS	Human (4.8, 8, 14)	NE	N/A nm, 1000 ms	mRNP nuclear export occurs faster than nucleoplasmic diffusion. Transcription, transport, and export occur between 5 and 40 min.	[62]
Super-registration microscopy	Mouse embryo fibroblasts	β-Actin (3.3)	NE	25 nm, 20 ms	mRNA nuclear export includes a three-step process composed of docking, transport, and release with durations at ~80, 5–0, and ~80 ms respectively. mRNA movement is not limited to one direction within the NPC, and not all nuclear pores observed were equally active in mRNA export.	[63]
Light sheet microscopy	*C. tentans* salivary gland	*C. tentans* (Up to 40)	NE	10 nm, 20 ms	~25% of mRNPs successfully export through the NPC. Export time was determined to be between 65 ms to several seconds. With a dwell time of ~55 ms, Dbp5 interacts with the NPC most frequently from the cytoplasmic side.	[64]
SPEED microscopy	HeLa	Firefly Luciferase (3.3)	NPC	8 nm, 2 ms	~36% of mRNPs interacting with NPC successfully complete the export process. During their ~12 ms transport time, mRNPs adopt a fast-slow-fast diffusion pattern while interacting with the periphery of the NPC and rarely enter the central axial conduit reserved for passive diffusion revealed by a 3D reconstruction of the export route for mRNPs through the NPC in live cells.	[65]

Abbreviations: NE, nuclear envelope; N/A, not available; NPC, nuclear pore complexes; mRNP, messenger ribonucleoprotein particles; SPEED, single-point edge-excitation sub-diffraction.

## 3. Various Single-Molecule Techniques Applied to Map Messenger Ribonucleoprotein Particles (mRNP) Export through the NPC

With yellow fluorescent protein (YFP) tagged mRNA transcripts and mCherry-labeled NPCs, Mor *et al.* [62] employed wide-field epi-fluorescence microscopy to track nuclear export of single mRNP molecules containing various larger mRNA constructs (14, 8, and 4.8 kb) in living cells (Table 1). Particularly, mRNPs were imaged via YFP fused MS2 coat proteins specific to different forms of human dystrophin mRNAs, transcribed in transfected U2OS cells. Taking advantage of the large field of illumination provided by wide-field imaging, many single mRNPs were simultaneously visualized in the nuclear as well as cytoplasmic portions of the cell. By quantifying single mRNPs in these cellular locales over time, a timeframe for cumulative transcription, transport and export of mRNA was estimated between 5 and 40 min with concentrations in the nucleoplasm remaining approximately constant due to continued transcription (Table 1). The temporal accuracy provided by the charge-coupled device (CCD) camera incorporated into the optical setup allowed for a frame rate of 1s per frame. Single mRNPs were recorded to cross the fluorescent NE as transported from the nucleus to the cytoplasm resulting in a transport rate fifteen times faster than simple diffusion in nucleoplasm, backing up the model for facilitated transport of mRNPs through the NPC [62,66].

With further modifications of wide-field epi-fluorescence setups, super-registration microscopy has been achieved by spatially registering two sources of emission fluorescence separated by a dichroic filter onto two pre-aligned CCD cameras. Thus, it was possible to image the mCherry-labeled NPCs and YFP-tagged endogenous β-actin mRNPs due to the super-registration set up. To achieve the labeling of native *β-actin* mRNA, the experimental design incorporated a transgenic mouse cell line in which YFP–MS2 proteins associated with endogenous β-actin mRNAs providing the advantage of imaging mRNA within its natural cellular environment. With a spatial and temporal resolution of 25 nm and 20 ms, super-registration microscopy revealed a three-step model for mRNA export through the NPC within a total transport time of ~180 ms, composed of docking (~80 ms), transport (5–20 ms) and release (~80 ms) processes (Table 1). Positional data of detected single mRNPs relative to the NPC center resulted in more interaction sites at the nucleoplasmic and cytoplasmic faces of the NPC compared to the central channel. One dimensional (1D) diffusion coefficient of mRNPs were determined from this data favoring a model in which nuclear export of mRNPs occurs faster during the central channel than the docking and the release steps on the nuclear and cytoplasmic regions of the NPC. Additionally, it was determined that not all NPCs are equally active in exporting mRNPs, and bidirectional movement of mRNPs is possible within the NPC [63].

Soon after, light sheet microscopy (LSM) was exploited to image single mRNP molecules exporting through the NPC in live cells. In contrast to wide-field epi-fluorescence microscopy and super-registration microscopy, the excitation laser beam in LSM penetrates the sample perpendicular to the direction of detection. The laser illumination profile is modified to produce a thin light sheet that can be used for optical sectioning. Since only a narrow strip of sample is illuminated at one time, photobleaching and phototoxic effects are minimal outside of the focused region and the lack of unwanted background fluorescence results in good signal to noise ratio [67,68,69,70]. mRNA nuclear export in a *Chironomus tentans* salivary gland cell system was mapped by LSM with a spatiotemporal resolution of ~10 nm and 20 ms (Table 1). In detail, endogenous individual mRNP was tracked via an Alexa-fluor-647-hrp36 fusion protein microinjected into the cells. Hrp36 is a ribonucleoprotein that associates with mRNA during the packing process resulting in mRNPs and remains associated with mRNPs until the start of translation. When fluorescently labeled, Hrp36 provided a tag thought to be less disturbing to the natural export machinery than MS2 coat proteins and also allowed different-sized endogenous mRNA to be imaged. To act as a reference point for tracking nucleocytoplasmic transport, the NE was illuminated by Alexa-fluor-546 labeled NTF2, a transport receptor known to facilitate RanGDP import into the nucleus [71,72,73]. By tracking these single fluorescent mRNPs in relation to labeled NE, it was determined that ~25% of mRNPs interacting with the NE successfully exported from the nucleus into the cytoplasm and the rest: ~75% returned to the nucleus. Nuclear export time was found to range from 65 ms to several seconds, suggesting different dwell times needed for tagged mRNPs of various sizes to transport through the NPC. In this work, Dbp5, a helicase necessary for mRNA export [54,74,75] was also fluorescently labeled and visualized in live cells, where it was seen to interact most frequently on the cytoplasmic sides of NPCs for a time of ~55 ms [64].

More recently, our lab has developed and applied an innovative super-resolution imaging technique, termed as single-point edge-excitation sub-diffraction (SPEED) Microscopy, to elucidate the export kinetics and map the three-dimensional (3D) spatial export route for firefly luciferase mRNPs in live NPCs with an unprecedented spatiotemporal resolution of 8 nm and 2 ms (Table 1) [23,65,76,77]. Our methodology includes two critical steps. First, we set out to obtain two-dimensional (2D) single-molecule trajectories of mcherry-MS2 associated mRNPs by specifically illuminating a single GFP-labeled NPC and tracking single mRNP molecules through this NPC in live cells. Second, we further recover the inherent 3D spatial distribution of mRNPs in the NPC by developing 2D to 3D deconvolution algorithms [23,77].

**Figure 2:**
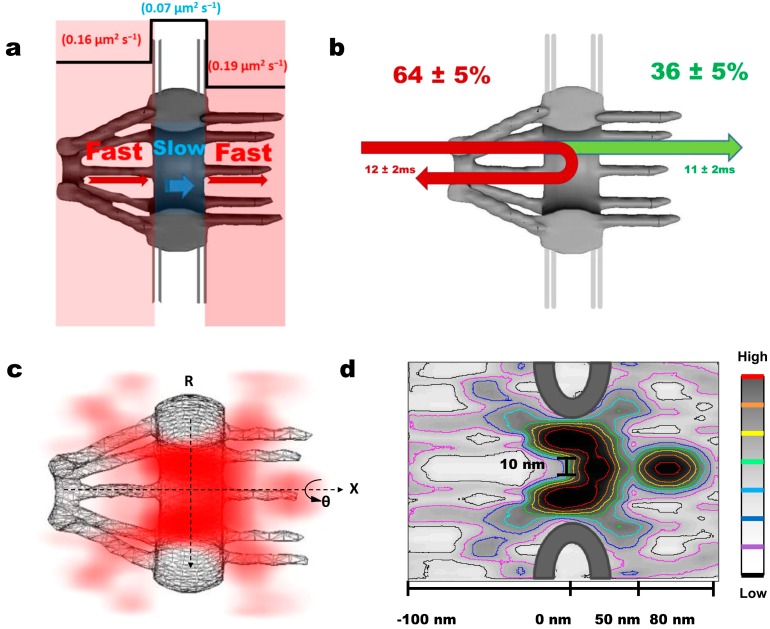
The major new features in the dynamic nuclear export process of messenger ribonucleoprotein particles (mRNPs) revealed by single-point edge-excitation sub-diffraction (SPEED) microscopy. (**a**) Cartoon representation of the fast-slow-fast diffusion pattern that mRNA takes through the NPC; (**b**) Diagram shows the abortive and the successful transport events. Green depicts ~36% of recorded single mRNPs that successfully traveled through the NPC and reached the cytoplasm during their ~11 ms transport time. Depicted in red are the abortive events ~64% and the associated transport time of ~12 ms; (**c**) 3D probability density map reveals the nuclear export route through the NPCs in live cells. mRNP 3D positional data (red) is plotted over a cutaway schematic representation of NPC structure. (Deeper shading represents higher density). mRNPs translocate through the periphery of the NPC while rarely occupying a central axial channel before dissociation occurs on the cytoplasmic side; (**d**) Contour plot of mRNP export through the NPC highlighting the unoccupied axial channel (from −100 to 20 nm) with a diameter of 10 nm at the narrowest waist and the dissociating region, where mRNA invades the central axial channel at the end of its export pathway (from 50 to 80 nm). Color bar indicates spatial density from low to high.

For the first aim, we shifted two overlapped excitation laser beams differing in wavelength off the center of the objective to create two superimposed inclined illumination PSFs (iPSFs) in the focal plane; one iPSF is to capture only a single GFP-labeled NPC at the equator of NE and the other is to track single mRNP molecules interacting with this illuminated NPC. The received fluorescent spot of a single NPC was then fit to a 2D elliptical Gaussian function to obtain the centroid of the NPC with a localization precision of 1–3 nm [77]. A frame rate of 500 frames per second enabled us to capture any transient interactions between the mRNPs and the NPC. We found the dwell time of mRNPs through the NPCs to be approximately 12 ms, ten-fold faster than previously reported data [63,65]. The difference is partially due to the different detection speeds and the distinct localizing approaches of single NPCs that our two groups used [65]. During the ~12 ms transport time, ~36% of mRNPs interacting with the NPC successfully travelled through the NPC to reach the cytoplasm. The remaining ~64% of transport events were abortive and the mRNPs were returned back to the nucleoplasm (Figure 2b) [65]. This transport efficiency is consistent with previous results [63,64] but deviates from the efficiency rate (~50%) recorded for Tap-p15 and other transport receptors indicating a stricter selective regulation for mRNPs at the NPC [23,65,76,77]. To further elucidate the activity of these successful and abortive mRNP transport events, single molecule trajectories were superimposed over an NPC schematic. Interestingly, ~80% of abortive mRNPs never reached the cytoplasmic face of the NPC with their deepest penetration reaching only the nuclear and central NPC sub-regions (−120 to 20 nm). In contrast, mRNPs that successfully translocated the NPC unavoidably arrived into the cytoplasmic sub-region (20–120 nm) whereupon dissociation was seen to occur mainly within the range of 50–80 nm on the cytoplasmic side of the NPC (Figure 2d). Apparently, these data support that major selection of mRNPs happens most likely on the nucleoplasmic side and within the central channel of the NPC [65].

Relating positional data with the ~12 ms transport time for mRNPs, we estimated the dwell time of mRNPs in each NPC sub-region to be: ~5.5 ms on the nucleoplasmic side, ~1.6 ms for the central region, and ~4.9 ms for the cytoplasm side [65], which indicates an interaction time ~3 times longer at the nucleoplasmic and cytoplasmic faces compared with the central channel of the NPC. This ratio of dwell times for mRNPs agrees well with the results from Grünwald and Singer, though there is almost a ten-fold difference between our measurements of total nuclear export times for mRNPs [63,65]. Noteworthy, we further found that the longer residence times of mRNPs on both sides of the NPC do not suggest mRNPs move slower on either side than in the center. Instead, from hundreds of obtained single mRNP trajectories, the diffusion coefficient of mRNP was obtained in each sub-region of the NPC: ~0.16 µm^2^/s on the nucleoplasmic side, ~0.07 µm^2^/s in the central scaffold region, and ~0.19 µm^2^/s on the cytoplasmic side, revealing a fast-slow-fast diffusion pattern adopted by mRNPs to export through the NPC (Figure 2a). This diffusion pattern brings support for the models favoring an entropic barrier in the central channel of the NPC due to geometric constraints and higher spatial density of FG filaments [42,44,45,65].

In the second step, by virtue of the cylindrical topology of the NPC, 2D positional data recorded from single mRNPs interacting with the NPCs were converted to their cylindrical 3D coordinates using deconvolution algorithms, which eventually resulted in 3D probability density maps for mRNPs in the NPC (Figure 2c,d). A 3D view of the real-time mRNP export route revealed for the first time that (i) mRNPs seldom present in the central axial channel that is reserved for small molecules (<40 kDa) to passively diffuse through; and (ii) Instead, mRNPs translocate through the periphery of the NPC prior to dissociating within the region of (50–80 nm) on the cytoplasmic side, at which point mRNPs have a low probability of returning to the nucleoplasm and start extending into the previously unoccupied axial channel (Figure 2d) [65,76,77]. This agrees with the proposition of a narrow release site located on the cytoplasmic side of the NPC [63]. Moreover, this dissociating region may be where the DEAD box helicase Dbp5 and its cofactor Gle1 function to dissociate the Tap-p15/mRNP complex from Nup214 and/or remodel the mRNP complex to facilitate directionality to complete the nuclear export process [64,74,77,78,79]. Another potential player is RanBP2 localizing around the cytoplasmic side of the NPC, which is believed to be involved in the dissociation process as well [80].

## 4. Conclusions and Perspective

Single-molecule tracking and super-resolution microscopy imaging finally brought the detailed dynamics of mRNP nuclear export into focus. Many new features escaped from conventional fluorescence microscopy and EM like export time, export efficiency, diffusion coefficients and 3D export route for mRNPs have been revealed by the technical advances and summarized in this review paper. We expect at least two major developments regarding the NPC and RNA biology in the near future. One is to expand current high-resolution fluorescence microscopy imaging approaches to investigate nucleocytoplamic transport of other types of RNAs, such as rRNA, micro RNA and virus RNA, as well as perform comparative studies on mRNA regulated by different transport cofactors following possible distinct selective mechanisms. Comparisons between nuclear transport behaviors of these RNAs would provide more insight into other critical questions including the role the NPC plays in the quality control of RNAs. The other is to correlate super-resolution light microscopy with other high-resolution detection approaches, such as EM and atomic force microscopy, to further provide a more comprehensive understanding of RNA nuclear export in both function and structure.
